# Genotype-flexible plant genetic transformation: advances and prospects

**DOI:** 10.3389/fpls.2026.1786813

**Published:** 2026-03-11

**Authors:** Yajie Guo, Mengyao Li, Mengtian Liu, Huiyun Liu

**Affiliations:** College of Agronomy and the State Key Laboratory of Wheat and Maize Crop Science, Henan Agricultural University, Zhengzhou, Henan, China

**Keywords:** developmental regulators, gene editing, genetic transformation, genotype-flexible dependence, plant

## Abstract

Genetic transformation of elite crop varieties remains limited by genotype-specific recalcitrance and dependence on tissue culture. This review provides a comprehensive analysis of current transformation platforms and emerging strategies to overcome these bottlenecks. We examine traditional *Agrobacterium*-mediated and biolistic methods, then critically assess tissue culture-free approaches including floral-based delivery, cut-dip-budding, *in planta* injection, and viral vector-mediated transformation systems. A major focus is the deployment of developmental regulators—*WUS/BBM*, *GRF-GIF* chimeras, *DOF* transcription factors, *LAX1*, *PLT*, and *WIND1*—that enhance regeneration efficiency across genotypes. We detail their molecular mechanisms, from chromatin remodeling and auxin gradient establishment to wound-responsive cell reprogramming. Importantly, we address the pleiotropic developmental costs of DR misexpression and review precision control technologies, including promoter optimization and auto-excision systems, that enable transient DRs activity during regeneration while ensuring normal plant development. We propose a roadmap for integrating these advances to achieve genotype-flexible, high-throughput transformation applicable to molecular design breeding.(1)

## 1 Introduction

Plant genetic transformation entails the stable and heritable integration of exogenous DNA—including synthetic expression cassettes or cross-kingdom sequences—into the nuclear or organellar genome of plants, yielding transgenic lines with constitutive expression the introduced traits (Bélanger et al., 2024; Bennur et al., 2025). By overcoming the meiotic barriers inherent to conventional hybridization, this technology enables the rapid and targeted transfer of beneficial alleles across taxonomic boundaries, shortening crop breeding cycles from decades to months. Recent advances in CRISPR nucleases and genotype-flexible regeneration protocols have further expanded the technical versatility and commercial applicability of plant genetic transformation (Law et al., 2022). Nevertheless, the transformation process is constrained by two interconnected bottlenecks: the efficient delivery of exogenous DNA into recipient plant cells and the subsequent regeneration of fertile transgenic plants (Xu et al., 2022).

Gene delivery strategies for plant genetic transformation are broadly classified into direct and vector-mediated approaches. Direct delivery methods, such as biolistics (particle bombardment), microinjection, ultrasonication, and nanocarrier-facilitated internalization, physically penetrate the plant cell wall to deliver exogenous DNA into the cytoplasm or nucleus. In contrast, vector-mediated (indirect) strategies exploit the natural horizontal gene transfer machinery of *Agrobacterium tumefaciens* or engineered viral vectors to mediate DNA integration into the host genome (Su et al., 2023; Wang et al., 2025a).

**Figure 1:**
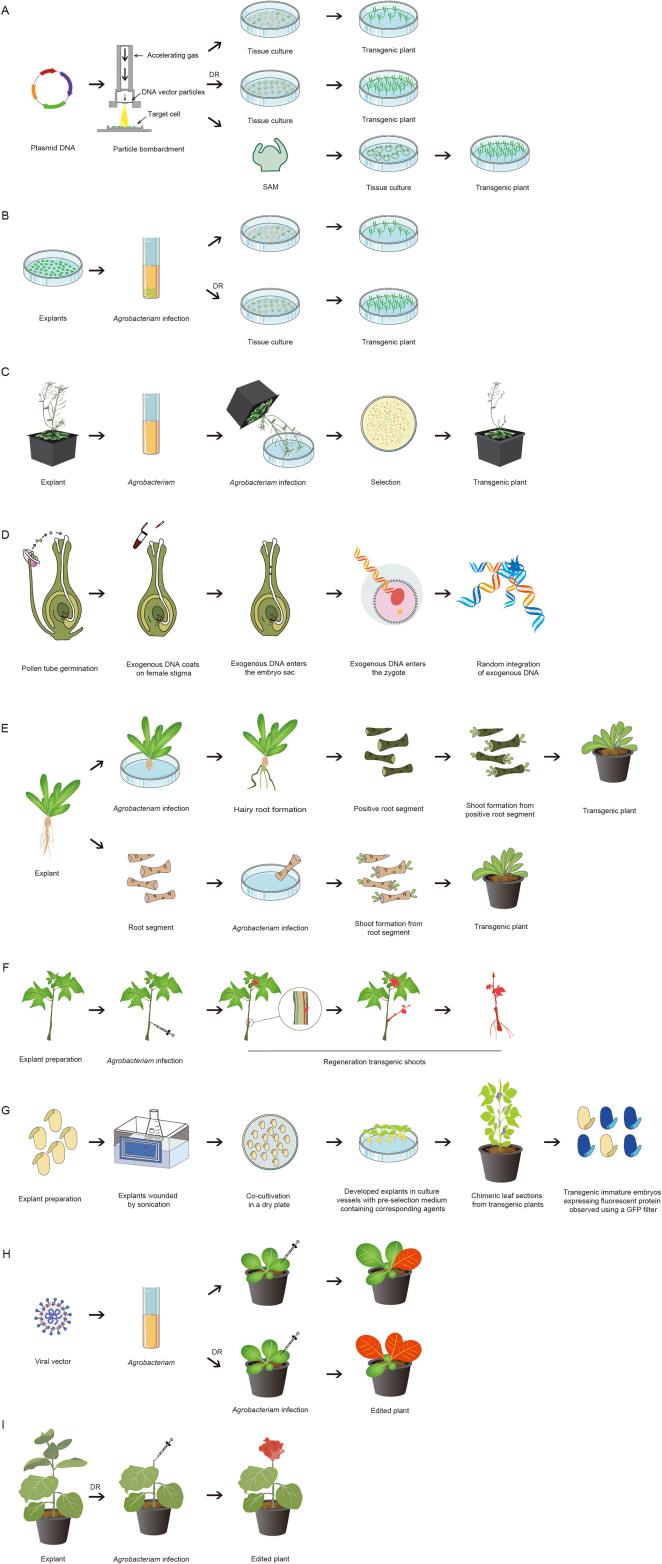
Schematic diagram of plant genetic transformation methods. **(A)** Particle bombardment of plant genetic transformation; **(B)** *Agrobacterium*-mediated plant genetic transformation; **(C)** Floral dip method; **(D)** Pollen-tube pathway method; **(E)** Cut-dip-budding (CDB) method; **(F)** Regenerative activity-dependent in planta injection delivery (RAPID) system; **(G)** Genotype-independent fast transformation (GiFT) system; **(H)** Virus-mediated plant genetic transformation; **(I)** In planta injection-mediated transformation systems, DR (Developmental regulators).

Among these approaches, biolistic and *Agrobacterium*-mediated transformations are the most widely utilized techniques in plant biotechnology, yet they generate markedly distinct genomic integration profiles (**Figures 1A, B**). Particle bombardment typically results in fragmented, multicopy transgene insertions at random genomic loci, which is strongly associated with transgene silencing, chromosomal instability, and unstable trait expression (Quiroz et al., 2024). By contrast, *Agrobacterium*-mediated transformation facilitates the delivery of intact, single-copy T-DNAs (transfer DNAs) with minimal sequence rearrangement, ensuring robust and stable transgene expression and negligible false-positive events across a broad range of explant types (Wu et al., 2024). Beyond DNA delivery efficiency, successful plant genetic transformation is inherently dependent on the reactivation of cellular totipotency—a core prerequisite for regenerating whole transgenic plants from transformed single cells or explants. Recently, Tang et al. (2025) demonstrated that *Leafy cotyledon2* (*LEC2*), a master regulator of plant embryogenesis, physically interacts with *Speechless* (*SPCH*), a key determinant of stomatal lineage specification, to induce localized auxin biosynthesis in individual somatic cells. This targeted auxin burst drives the epigenetic and transcriptional reprogramming of differentiated somatic cells into pluripotent stem cells with the capacity to regenerate whole plants. Collectively, these findings established a mechanistic framework for optimizing regeneration protocols, particularly for genotypes that are traditionally recalcitrant to transformation (Tang et al., 2025).

Conventional *Agrobacterium*-mediated transformation typically employs sterile explants (e.g., seedlings, immature embryos, cotyledons, epicotyls, hypocotyls, or callus) that require culture under stringent *in vitro* conditions. This approach demands labor-intensive, genotype-specific optimization of regeneration media and protocols, and is often associated with low or inconsistent transformation efficiency (Bélanger et al., 2024). In contrast, non-tissue-culture methods harness the innate regenerative capacity of plants, enabling the formation of transgenic organs and whole plants without sterile *in vitro* culture steps (Hao et al., 2024) (**Figures 1C–I**). Despite their advantages, the efficacy of non-tissue-culture approaches has been demonstrated primarily in transformation-amenable species (e.g., cereals, legumes, and tuber crops), with limited success in recalcitrant species, woody perennials, and elite commercial cultivars—all of which exhibit reduced transformation competence compared to model genotypes (Mei et al., 2024; Zhong et al., 2024). Thus, while non-tissue-culture transformation strategies hold great promise, they remain constrained by species-specific biological characteristics and have not yet achieved the universal applicability required for large-scale agricultural deployment.

A critical advancement in addressing these limitations is the deployment of developmental regulators (DRs), including *Wuschel* (*WUS*), *Baby boom* (*BBM*), *Growth-regulation factor 4* (*GRF4*), *GRF-interaction factor 1* (*GIF1*), *TaWOX5*, *TaLAX PANICLE1* (*TaLAX1*), *Wound induced dedifferentiation 1* (*WIND1*), and *Plethora* (*PLT*). These regulators have been shown to significantly enhance plant regeneration frequency across both direct or indirect transformation systems (Lowe et al., 2016; Debernardi et al., 2020; Lian et al., 2022; Wang et al., 2022). By promoting cell proliferation and epigenetic reprogramming of somatic cells, DR-mediated strategies provide a viable pathway to overcome genotype-dependent transformation recalcitrance, though their seamless integration with non-tissue-culture DNA delivery systems remains an active area of investigation and optimization.

In this review, we provide a comprehensive overview of current plant genetic transformation platforms and synthesize recent advances in DR-mediated regeneration and culture-free transformation strategies. We also propose a roadmap for the development of rapid, high-throughput, and genotype-flexible transformation protocols applicable to diverse plant species. To ensure conceptual clarity, we adopt precise terminology for transformation systems: genotype-independent refers to systems functional across diverse genotypes or elite varieties within a single species (e.g., the GiFT system in multiple soybean cultivars) (Zhong et al., 2024); genotype-flexible describes methods applicable across related species with shared biological features (e.g., BBM/WUS2 systems across the Poaceae family) (Lowe et al., 2016; He et al., 2025); and genotype-free transformation—implying universal applicability across all plant taxa regardless of genetic background—remains a theoretical ideal not yet realized with current technologies.

## 2 Traditional plant genetic transformation system

### 2.1 Particle bombardment-mediated plant genetic transformation

Particle bombardment is a physical DNA delivery technique that dispenses with biological vectors, first developed by Sanford and colleagues and subsequently established as a cornerstone of plant genetic modification research (Klein et al., 1992). This approach relies on high-velocity metal particles (typically gold or tungsten microcarriers) to ferry exogenous DNA constructs directly into plant cells: DNA is adsorbed onto the surface of metal particles, which then penetrate the rigid plant cell wall and plasma membrane via the driving force of high-pressure gas or gunpowder detonation. Following intracellular delivery, the exogenous DNA either integrates into the host genome for stable and heritable expression or undergoes transient expression without genomic integration (**Figure 1A**).

Particle bombardment exhibits particular efficacy in monocotyledonous species (e.g., rice, maize, and wheat), which are often recalcitrant to *Agrobacterium*-mediated transformation (Vasil et al., 1992). A notable advancement was reported by Hamada et al. (2017), who utilized meristematic tissues of mature wheat embryos as transformation recipients to generate stable transgenic plants, completely circumventing the laborious steps of callus induction and subsequent plant regeneration (**Figure 1A**). Building on this breakthrough, subsequent studies have delivered CRISPR/Cas9 and CRISPR/Cas12a genome editing reagents via particle bombardment, establishing efficient, stable, and genotype-independent gene editing systems for the production of transgene-free wheat lines (Kumagai et al., 2022; Luo et al., 2025). Beyond cereals, this method has also been successfully applied to peanut embryo transformation: by leveraging CRISPR/Cas9 to knockout the *AhFAD2–1* gene, researchers obtained high-oleic-acid peanut mutants devoid of transgenic traces, validating particle bombardment as a rapid strategy for the quality improvement in oilseed crops (Shi et al., 2025).

Nonetheless, particle bombardment (gene gun)-mediated transformation is not without limitations, particularly in comparison to *Agrobacterium*-mediated approaches. Key drawbacks include cumbersome operational procedures, elevated experimental costs, and inherent constraints on the size of deliverable exogenous DNA fragments. Moreover, this method is associated with random genomic integration, high transgene copy numbers, and a heightened propensity for transgene silencing—all of which can compromise the stability and predictability of transgene expression in transgenic plants (**Table 1**).

**Table 1:** Comparison of established and emerging plant genetic transformation platforms.

Platform	Tissue culture requirement	Heritability	Regeneration requirement	Key advantage	Key limitation
Particle bombardment	Required	Stable or transient	Required	No host specificity limitation; broad applicability to cell/tissue types	DNA fragmentation
*Agrobacterium*-mediated	Required	Stable	Required	Low copy number integration; high stability and heritability	Genotype dependency
Floral dip	Not required	Stable	Not required	Tissue culture-free; easy operation	Extreme species specificity; low and unstable transformation efficiency
Pollen-tube pathway	Not required	Controversial	Not required	Tissue culture-free; genotype-independent	Poor reproducibility; low transformation efficiency
Cut-dip-budding	Hairy root induction	Stable	Required planta budding	No sterile culture needed	Restricted to species with root-shoot transition competence
RAPID	Not required	Stable	Partial required (direct organogenesis)	Tissue culture-free; genotype-flexible	Vegetative propagation dependency
GiFT	Not required	Stable	Selection-based, not required tissue culture	Tissue culture-free; genotype independence	Limited to seed-propagated crops; herbicide selection requirement; seed germination synchronicity dependence
Conventional VIGE system	Required	Transient/non-heritable	Required	High copy number	Cargo size limit; transient expression; viral host specificity
Newly VIGE system	Not required	Heritable	Not required	Tissue culture-free	Cargo size limit; viral host specificity

### 2.2 *Agrobacterium*-mediated plant genetic transformation

*Agrobacterium*-mediated plant transformation capitalizes on the innate horizontal gene transfer machinery of the soil-dwelling bacterium *Agrobacterium tumefaciens* to deliver exogenous genetic material into the plant nuclear genome (**Figure 1B**). It is widely regarded as one of the most reliable and efficient tools for plant genetic modification, with key advantages including low transgene copy number, stable heritable genomic integration, technical simplicity, low experimental cost, and the capacity to transfer large DNA fragments with minimal sequence rearrangements (**Table 1**). These features collectively reduce the risk of transgene silencing and facilitate downstream genetic analysis and trait validation, making the method of choice for both basic plant science research and applied crop genetic improvement (Huang et al., 2024).

A defining feature of *Agrobacterium tumefaciens* is its harboring of a tumor-inducing (Ti) plasmid, which encodes a discrete segment of DNA known as T-DNA. In its native state, the *bacterium* translocates the T-DNA into host plant cells, where it integrates stably into the plant genome and expresses oncogenic genes, ultimately triggering the formation of crown gall tumors. To harness this system for biotechnological applications, researchers have engineered the Ti plasmid by replacing these tumor-causing genes with target transgenes of interest. This genetic modification repurposes *Agrobacterium tumefaciens* as a highly versatile gene delivery vector, facilitating the precise and stable integration and subsequent expression of exogenous genes in recipient plant cells (Neelakandan et al., 2023).

In 1985, Horsch et al. pioneered the *Agrobacterium*-mediated leaf disk method, a breakthrough that enabled the successful generation of transgenic tobacco plants. This seminal protocol was subsequently adapted for the production of transgenic lines in species such as *Ipomoea nil* (dwarf morning glory) and tomato, validating its broad applicability across dicotyledonous plants (Horsch et al., 1985). With iterative advancements in vector design and transformation system optimization, *Agrobacterium*-mediated genetic transformation has transcended its initial dicot bias and emerged as a viable tool for engineering monocotyledonous crops, including rice, maize, wheat, barley, and foxtail millet (**Figure 1B**). A landmark achievement occurred in 1996, when Ishida et al. employed the *Agrobacterium tumefaciens* strain LBA4404 to transform maize embryos—the first successful generation of transgenic maize via this approach (Ishida et al., 1996). The following year, Cheng et al. (1997) extended this progress to wheat, producing transgenic wheat plants through the *Agrobacterium*-mediated transformation of wheat embryos, and researchers have since established highly efficient *Agrobacterium*-mediated transformation systems using immature embryos of the spring wheat cultivar Fielder as explants (Ishida et al., 2015). Although this method has facilitated the genetic modification of multiple wheat varieties, its utility remains constrained by recalcitrance in certain genotypes with poor regeneration capacity (e.g., Jimai 22, Sumai 3, Ningchun 4, Aikang 58, and Xinong 979) (Ishida et al., 2015). More recently, Wang et al. (2025b) developed a universal, stable, and high-efficiency genetic transformation system tailored to sugarcane. Their strategy entailed the induction of embryogenic calli from tender sugarcane core leaves, which were then deployed as recipient explants for *Agrobacterium*-mediated transformation. By implementing continuous herbicide selection across three critical developmental stages—callus propagation, shoot differentiation, and seedling rooting—they achieved robust proliferation of transformed resistant calli, efficient shoot regeneration, and stable rooting of transgenic plantlets, thereby overcoming several key bottlenecks in the genetic engineering of sugarcane (Wang et al., 2025b).

## 3 Emerging tissue culture-free transformation platforms

The development of plant transformation technologies that reduce or eliminate reliance on elaborate *in vitro* tissue culture represents a major advance in crop genetic improvement, though their broader applicability remains contingent on overcoming species-specific biological constraints. Conventional *Agrobacterium*-mediated methods (e.g., the leaf disk transformation protocol) often depend on callus induction—a step that is recalcitrant or unfeasible in many plant taxa—and exhibit pronounced variability in efficiency across species and genotypes, thereby limiting scalable genetic engineering. In response, the pursuit of tissue culture-free and minimal-tissue-culture transformation approaches has catalyzed innovative methodologies that leverage the innate regenerative capacity of plant shoots, roots, and reproductive tissues. Here, we review these methodologies—including floral-based delivery (floral dip and pollen-tube pathway), cut-dip-budding (CDB), in planta injection (RAPID and GiFT), and viral vector-mediated transformation—critically assess their efficacy across diverse crop systems, and identify strategies to overcome their limitations in elite cultivars and recalcitrant species.

### 3.1 Floral tissue-based transformation

The floral dip method is a seminal breakthrough in tissue culture-free plant transformation. Originally described for *Arabidopsis thaliana* by Bechtold and Pelletier (1993) and later optimized by Clough and Bent (1998), this approach involves dipping flowering inflorescences into an *Agrobacterium tumefaciens* suspension containing a suitable surfactant (Bechtold and Pelletier, 1993; Clough and Bent, 1998). The bacteria directly enter ovule cells through natural openings, delivering transgenes into the plant’s reproductive tissues and yielding transgenic seeds without the need for intervening *in vitro* tissue culture steps (**Figure 1C**). Renowned for its operational simplicity and rapidity, the floral dip method has revolutionized genetic manipulation in *Arabidopsis* and has been successfully adapted to a range of dicotyledonous species. It has also been extended to monocot models species, such as *Setaria viridis* (Kaur and Devi, 2019; Bélanger et al., 2024). However, despite its broad utility, application of the floral dip method in economically important monocot crops (e.g., wheat and rice) has thus far achieved only low and unstable transformation efficiencies, underscoring the need for targeted technical refinements to expand its applicability across angiosperm taxa (**Table 1**).

Parallel research efforts have explored alternative *in planta* floral transformation strategies, with the pollen-tube pathway method being the most well-characterized. First reported in cotton by Zhou et al. (1983), this method involves introducing foreign DNA into the style of newly pollinated flowers, where it is transported to the fertilized ovule via growing pollen tubes (Zhou et al., 1983) (**Figure 1D**). The pollen-tube pathway method offers distinct advantages for plant genetic transformation: (i) complete elimination of *in vitro* tissue culture, thereby avoiding somaclonal variation and reducing technical complexity; (ii) inherent genotype independence, enabling the transformation of diverse species and recalcitrant commercial cultivars; (iii) operational simplicity and low cost, requiring no specialized equipment and allowing implementation in field or greenhouse conditions; and (iv) utilization of the plant’s natural reproductive machinery, with pollen tubes serving as biological vectors to deliver exogenous DNA directly to the zygote or early embryo. A similar approach applied to rice enabled the rapid recovery of transgenic seeds, although its utility has been constrained by low transformation efficiency and inconsistent results. Nonetheless, recent advances highlight its untapped potential: for instance, efficient transgenic peanut production was achieved through *Agrobacterium*-mediated pollen tube transformation via simple style injection (Zhou et al., 2023). In maize, Yang et al. (2017) reported a method in which pollen grains were pretreated with sucrose and aeration at 4 °C prior to sonication-assisted gene delivery, which significantly improved pollen viability and subsequent transformation efficiency. Successful reporter gene expression was observed in pollen tubes, developing embryos, and stable transgenic maize lines (Yang et al., 2017). Collectively, these developments highlight ongoing innovation in tissue culture-free floral transformation; while the floral dip method remains a cornerstone for many species, the pollen-tube pathway offers a promising avenue for the genetic engineering of major cereal and legume crops (**Table 1**).

### 3.2 Cut-dip-budding approaches

A pivotal development in tissue culture-free transformation is the CDB method, which harnesses the natural Ri plasmid of *Agrobacterium rhizogenes* and is based on the active *in planta* regeneration capacity of excised shoot cuttings. In this approach, *Agrobacterium* rhizogenes is delivered to plant meristems via local injection to induce transgenic nascent tissues, and stable transgenic plants are obtained by subsequent vegetative propagation of the positive transgenic nascent tissues. The foundational CDB protocol, established for the sweet potato (*Ipomoea batatas*), involves inoculating wounds at the rhizome junction with *Agrobacterium rhizogenes*, directly inducing regenerative buds on stems under nonsterile conditions and thereby bypassing sterile *in vitro* tissue culture (**Figure 1E**). This system was successfully used for CRISPR/Cas9-mediated editing of starch synthase genes in sweet potato, yielding transgenic lines with modified starch composition (Cao et al., 2023a). In 2023, Zhang et al. introduced a simple one-step CDB method that involves inoculating sweet potato shoot cuttings with *Agrobacterium rhizogenes* and planting them directly in soil to generate transgenic tubers and shoots (Zhang et al., 2023). Subsequently, an enhanced, simplified version (ES-CDB) was developed to eliminate the hairy root cultivation phase, which significantly shortened the transformation cycle (**Figure 1E**) (Cao et al., 2023b). Similar success has been reported with Russian dandelion (*Taraxacum kok-saghyz*) and citrus, demonstrating the potential of *Agrobacterium rhizogenes-*mediated CDB transformation for woody tree species (Ramasamy et al., 2023). Additionally, this system has been successfully adapted for chickpea (*Cicer arietinum*) to edit the *Sal1* gene, yielding drought-tolerant lines with doubled root branching without ever exposing plant tissues to *in vitro* culture medium (Abdallah et al., 2024). While the CDB method represents a breakthrough in simplifying plant genetic transformation by eliminating tissue culture requirements, its primary limitation is the restriction to species capable of root suckering, combined with variable transformation efficiency and species-specific optimization requirements (**Table 1**). These factors currently prevent it from being a truly universal transformation system applicable to all plant species (Hoerster et al., 2020; Cho et al., 2022).

### 3.3 *In planta* injection delivery transformation systems

The regenerative activity-dependent in planta injection delivery (RAPID) system exemplifies the optimization of physical DNA delivery for in planta transformation, wherein *Agrobacterium tumefaciens* suspensions are directly injected into the lower excised ends of stem segments of sweet potato (*Ipomoea batatas*) and bayhops (*Ipomoea pes-caprae*), or beneath the peridermal skin of potato (*Solanum tuberosum*) tubers (Mei et al., 2024). In this protocol, *Agrobacterium tumefaciens* is delivered to plant meristems cells to elicit the formation of transgenic nascent tissues (Zhong et al., 2025); stable transgenic lines are subsequently regenerated via vegetative propagation of these transformed meristematic tissues. Distinguished by its operational simplicity, high transformation efficiency (12.5–40% depending on species and genotype), and short turnaround time (3–10 weeks), the RAPID system has emerged as a practical tool for plant genetic engineering (**Figure 1F**) (Mei et al., 2024). For instance, in potatoes, the injection of disarmed *Agrobacterium tumefaciens* into the peridermal tissue surrounding tuber buds resulted in the regeneration of transgenic shoots with robust reporter gene expression and high-efficiency targeted gene editing (Mei et al., 2024). Analogously, direct injection of *Agrobacterium tumefaciens* into the meristematic zones of *Panax notoginseng* rhizomes and *Lilium regale* bulbs has enabled the generation of transgenic plants of these recalcitrant species (Deng et al., 2024). The RAPID approach achieves high transformation efficiencies by exploiting the innate regenerative capacity of vascular tissues—specifically the phloem meristematic zones—thereby eliminating the need for callus induction and shoot organogenesis on defined *in vitro* culture media (Mei et al., 2024). While RAPID represents a significant advancement in simplifying plant transformation by eliminating tissue culture requirements and avoiding *A. rhizogenes*-related developmental abnormalities, its primary limitation is the strict requirement for plants with strong active meristematic regeneration capacity and its current restriction to vegetatively propagated dicot species. The potential for chimeric transgenic plant formation and the need for species-specific injection and propagation optimization also present challenges for its broad agricultural application (**Table 1**).

Legume genetic transformation has historically been constrained by severe genotype-dependent recalcitrance, with conventional *Agrobacterium*-mediated methods typically achieving efficiencies of only 2–10% in elite varieties of soybean, common bean (*Phaseolus vulgaris*), and chickpea (Paz et al., 2006; Altpeter et al., 2016). Success has been largely restricted to model cultivars with exceptional *in vitro* responsiveness, which correlates poorly with agronomic value. This limitation is further exacerbated by the apparent incompatibility between *in vitro* tissue culture protocols and symbiotic nitrogen fixation competence, as prolonged *in vitro* manipulation disrupts the molecular circuitry required for rhizobial symbiosis in legumes. The genotype-independent fast transformation (GiFT) system addresses these constraints by circumventing elaborate *in vitro* tissue culture through direct meristem targeting at a precise developmental stage. Specifically, germinating soybean (*Glycine max*) seeds are inoculated with *Agrobacterium tumefaciens* at the developmental window when the cotyledonary node axillary meristem becomes accessible, followed by sublethal glufosinate selection to enrich for transgenic meristematic cells. This approach achieves transformation efficiencies of 1.0–93.8% across 11 diverse soybean varieties—including previously recalcitrant elite commercial cultivars—with an average efficiency of 24.5% (Zhong et al., 2024) (**Figure 1G**). Moreover, the 35-day timeline from seed imbibition to the establishment of T0 transgenic events represents a ~60% reduction compared to conventional cotyledonary node transformation methods (Zhong et al., 2024). A key advantage of in planta delivery systems (RAPID and GiFT) is the avoidance of tissue culture-induced somaclonal variation, but they remain limited to specific plant propagation modes: RAPID is restricted to species amenable to vegetative propagation (e.g., sweet potato, potato, bayhops) (Mei et al., 2024), while GiFT—though applicable to seed-propagated soybean—depends on precise seedling meristem targeting and requires herbicide resistance-based selection, with variable efficiency across elite commercial varieties (**Table 1**) (Zhong et al., 2024).

### 3.4 Virus-mediated plant genetic transformation

Virus-mediated plant genetic transformation is a versatile strategy that exploits genetically engineered plant viruses as vectors to deliver and express exogenous genetic elements in plant cells. Harnessing the intrinsic capacity of plant viruses to translocate nucleic acids across cell boundaries, these engineered viral vectors are endowed with the capabilities of replication, self-assembly, and systemic intercellular movement, thus enabling the efficient delivery of diverse exogenous molecules—including RNA interference reagents and genome editing components—to recipient plant cells (**Figure 1H**).

In a pioneering study, Ali et al. (2015) successfully integrated sgRNA expression cassettes into the *Tobacco rattle virus* (*TRV*) vector. Through *Agrobacterium-*mediated transient infection of Cas9-overexpressing tobacco plants, the method achieved efficient editing of the endogenous *PDS* (phytoene desaturase) gene (Ali et al., 2015). In a related investigation, Jiang et al. (2018) modified the *Beet necrotic yellow vein virus* (*BNYVV*) to express multiple sgRNAs, thereby facilitating simultaneous multiplex knockout of target genes in Cas9-overexpressing tobacco (Jiang et al., 2018). Separately, Hu et al. (2019) optimized the *Barley stripe mosaic virus* (*BSMV*) vector, which was subsequently deployed to achieve efficient knockout of endogenous genes in Cas9-overexpressing maize and wheat plants (Hu et al., 2019). However, conventional virus-induced gene editing (VIGE) systems have historically faced two major limitations: cargo size constraints that preclude the packaging of large CRISPR-Cas systems, and the requirement for pre-existing Cas9-transgenic recipient lines or the production of only transient, non-heritable editing events (Mikhaylova, 2025).

In recent years, the researchers have developed several innovative strategies to overcome these limitations achieve heritable genome editing via viral vectors. A notable breakthrough involved engineering viral vectors to harbor the *Flowering locus T* (*FT*) gene, which enabled the production of heritable mutant progeny following systemic viral infection of plant leaves. Ellison et al. (2020) first reported that *TRV* vectors delivering sgRNAs fused to *FT* could promote the cell-to-cell and long-distance mobility of sgRNAs into plant meristematic tissues—a prerequisite for transmitting editing events to the germline and subsequent generations (Ellison et al., 2020). This method was subsequently validated in *Arabidopsis thaliana* and tomato (Nagalakshmi et al., 2022; Lee et al., 2024). Extending this strategy to monocotyledonous species, two independent studies demonstrated that the *BSMV* vector could generate heritable genome-edited progeny in Cas9-overexpressing wheat lines (Li et al., 2021; Chen H. et al., 2022).

In 2025, two independent studies demonstrated the feasibility of viral delivery of ultra-compact genome editors to achieve heritable, transgene-free editing in plants: Weiss et al. utilized the ~400 amino acid TnpB nuclease (ISYmu1, 382 aa) in *Arabidopsis thaliana*, while Hu et al. employed the 422 amino acid AsCas12f in *Nicotiana benthamiana* and tomato (*Solanum lycopersicum*). Both systems bypass *in vitro* tissue culture and stable transgene integration, with editing events efficiently inherited in subsequent generations. AsCas12f offers broader PAM flexibility (5′-TTR-3′ vs. TnpB’s 5′-TTGAT-3′) and markedly higher editing efficiency (83.08% in *N. benthamiana* vs. 8.9% in *Arabidopsis* for TnpB). However, these ultra-compact nuclease viral delivery platforms are not yet universally applicable: viral host specificity, challenges in accessing meristematic tissues for efficient germline transmission, and variable systemic spread efficiency currently limit their demonstrated efficacy—with TnpB-TRV restricted to *Arabidopsis*, and AsCas12f-TRV limited to *Nicotiana* and tomato (Hu et al., 2025; Weiss et al., 2025; Mikhaylova, 2025). The extension of these viral delivery systems to major cereal crops and legumes remains an unmet experimentally goal (**Table 1**).

## 4 Exploration and application of plant developmental regulators

Plant regeneration induction constitutes a pivotal prerequisite for successful plant genetic transformation, as it enables the regeneration of whole plants from transformed single cells or explants. Extensive investigations into the regulatory genes governing plant regeneration have identified and cloned a suite of key genes implicated in somatic embryogenesis and organogenesis. Across both monocotyledonous and dicotyledonous plants, a subset of genes encoding DRs have been demonstrated to markedly elevate plant regeneration efficiency, a breakthrough that plays a critical role in accelerating the genetic transformation process of crop species (Jiang et al., 2024; Han et al., 2025).

### 4.1 *BBM* and *WUS* promote genetic transformation in plants

*BBM*, a member of the AP2/ERF transcription factor family, functions as a master regulator of plant somatic embryogenesis. *BBM* directly binds to the promoter regions of *LEC1*, *LEC2*, *FUS3*, and *ABI3*—the core components of the LAFL embryonic identity regulatory network—activating their expression through the recruitment of SWI/SNF chromatin remodeling complexes. This epigenetic reprogramming triggers a feed-forward regulatory cascade that establishes embryonic competence in differentiated somatic cells, enabling hormone-independent callus formation and plant regeneration (Jha and Kumar, 2018). *WUS*, encoding a homeodomain transcription factor expressed in the organizing center of the shoot apical meristem (SAM), functions as a master regulator of stem cell maintenance in the SAM. WUS promotes stem cell identity in adjacent meristematic cells while non-cell-autonomously repressing *CLAVATA3* (*CLV3*) expression, establishing a negative feedback loop that precisely constrains SAM stem cell niche size. Additionally, WUS directly represses A-type *ARABIDOPSIS RESPONSE REGULATOR* genes (*ARR5*, *ARR6*, *ARR7*, *ARR15*), dampening cytokinin signaling to prevent the premature differentiation of meristematic stem cells (Sarkar et al., 2007). In *Arabidopsis thaliana*, the *WUS* gene is indispensable for the establishment of embryonic apical meristematic tissues and the post-embryonic initiation of axillary meristems. In 2022, researchers isolated the *TaWOX5* gene from wheat callus tissues via sequence alignment with the *Arabidopsis WUS* protein. Functional characterization of transgenic wheat lines demonstrated that the overexpression of *TaWOX5* dramatically enhanced transformation efficiency, with a 2-10-fold increase in both model wheat genotypes and commercially cultivated varieties (Wang et al., 2022). Notably, *TaWOX5* exhibits a strict root tip-specific expression pattern, and its overexpression exerts no adverse effects on the development of aerial plant organs or root morphogenesis (Wang et al., 2022).

### 4.2 GRF-GIF chimeras enhance plant regeneration efficiency

GRF-GIF chimeric proteins are synthetic engineered factors generated by fusing a minimal N-terminal *GRF* fragment—containing the conserved WRC DNA-binding domain and QLQ protein-protein interaction domains—to the SNH transcriptional activation domain of *GIF1* (Debernardi et al., 2020). These synthetic chimeric proteins bypass the stoichiometric constraints of endogenous GRF-GIF complex assembly, potently activating cell proliferation via SWI/SNF-mediated chromatin remodeling and the upregulation of core cell cycle regulatory genes (*CYCD3;1*, *E2Fa*), thereby substantially enhancing plant transformation efficiency across diverse crop species. Studies have demonstrated that the chimeric gene *TaGRF4-TaGIF1* can significantly elevate transformation efficiency in wheat and other monocot species by modulating the expression of multiple genes associated with cell division and organogenesis (Debernardi et al., 2020). Beyond transformation efficiency enhancement, the TaGRF4-TaGIF1 chimeric protein also markedly shortens the wheat transformation cycle from 91 d to 56 d. When integrated with the CRISPR/Cas9 genome editing system, *TaGRF4-TaGIF1* further confers a substantial improvement in wheat gene editing efficiency (Debernardi et al., 2020).

### 4.3 *DOF* transcription factors boost regeneration efficiency

Although recent years have seen substantial advances in wheat genetic transformation technologies, regeneration recalcitrance remains a major constraint for a subset of elite wheat cultivars. In 2023, Liu et al. (2023b) integrated RNA sequencing (RNA-seq), assay for transposase-accessible chromatin with sequencing (ATAC-seq), and cleavage under targets and tagmentation (CUT&Tag) to dissect the transcriptional and chromatin accessibility dynamics during the early regeneration phase of immature embryo scutella derived from the wheat cultivar Fielder. Through this multi-omics integrative approach, they identified two DNA binding with one finger (DOF) family transcription factors, *TaDOF5.6* and *TaDOF3.4*, which markedly elevated callus induction frequency and genetic transformation efficiency across a panel of diverse wheat varieties.

DOF transcription factors are plant-specific regulatory proteins characterized by a conserved C-terminal C2C2 zinc finger DNA-binding domain that specifically recognizes the A/T-rich consensus sequence 5’-AAAG-3’ (Yanagisawa, 1995). Through diverse N-terminal regulatory domains, DOF proteins function as either transcriptional activators or repressors, modulating key plant developmental processes including seed maturation and germination, photoperiodic flowering, and hormone signaling (Yanagisawa, 2002). Liu et al. (2023) found that *TaDOF5.6* and *TaDOF3.4* function as core transcription factors in the wheat regeneration regulatory network, directly governing the expression of downstream genes implicated in cell cycle progression, cell proliferation, and lateral root development (Liu et al., 2023a).

### 4.4 Overexpression of *LAX1* enhances shoot regeneration efficiency in plants

*TaLAX1* encodes an auxin influx carrier belonging to the AUX/LAX family that facilitates polar auxin transport in wheat shoot meristems. By mediating cellular auxin uptake in meristematic cells, *TaLAX1* establishes localized auxin gradients that are critical for tiller bud initiation and panicle branching, thereby regulating wheat shoot architecture and grain yield components (He et al., 2021). In 2024, Yu et al. reported that the constitutive overexpression of *TaLAX1* significantly enhanced regeneration capacity, genetic transformation efficiency, and gene editing efficacy in several wheat genotypes. Subsequent molecular mechanism assays revealed that *TaLAX1* exerts its pro-regeneration effect by directly activating the transcription of *TaGRF4* and *TaGIF1*, which in turn promotes cytokinin accumulation and augments auxin signaling responses in explant tissues, ultimately facilitating efficient plant regeneration (Yu et al., 2024). Notably, the heterologous overexpression of *TaLAX1* orthologs also improved regeneration performance in maize and soybeans, highlighting the conserved function of this gene in regulating regeneration across multiple crop species (Yu et al., 2024).

### 4.5 *PLT* factors are involved in promoting somatic embryogenesis

Previous research has established that *WOX5* plays a pivotal role in modulating root meristem activity by recruiting *PLT* factors to drive cell differentiation processes in the root apical meristem (Ding and Friml, 2010). As members of the AP2/ERF transcription factor superfamily, PLT proteins exert profound regulatory effects on plant regeneration and organogenesis. Studies have demonstrated that *PLT3*, *PLT5*, and *PLT7* synergistically activate the expression of *PLT1* and *PLT2* in callus tissues, thereby enabling *de novo* generation of shoot progenitor cells from calli. Subsequent to this activation, these *PLT* factors induce the transcription of *Cup-shaped cotyledon 2* (*CUC2*), a key regulator of shoot meristem formation. Notably, *CUC2*, together with *PLT5*, participates in the plant tissue regeneration signaling cascade, where it promotes shoot primordium development and subsequent organogenesis (Kareem et al., 2015). Functional validation assays have further corroborated the conserved pro-regeneration function of *PLT5* across multiple plant species: for instance, *Agrobacterium*-mediated delivery of *PLT5* significantly enhanced callus formation and shoot regeneration at wound sites in snapdragon (*Antirrhinum majus*) and tomato; in rapeseed (*Brassica napus*), *PLT5* overexpression boosted shoot regeneration efficiency and genetic transformation rates; and in sweet pepper, it facilitated somatic embryo formation from explant tissues. Collectively, these findings confirm the conserved function of *PLT5* in improving genetic transformation efficiency across diverse plant taxa (Lian et al., 2022).

In 2025, Wittmer et al. established a pioneering hormone-free system for somatic embryogenesis and plant regeneration through the targeted screening of plant DRs. Their work identified that the synergistic expression of root stem cell regulators—*PLT* and *WOX* family factors—is indispensable for triggering somatic embryogenesis and subsequent plant regeneration from somatic cells. This discovery confirms the *PLT*/*WOX* module as the core regulatory hub governing regeneration induction in plants. Beyond this key finding, the study also elucidated the critical molecular pathways involved in transcriptome reprogramming and somatic embryogenesis initiation. Importantly, this hormone-independent regeneration system overcame the technical bottlenecks associated with regenerating recalcitrant crops (e.g., chili pepper), thus providing a novel strategy for advancing crop genetic engineering, breeding improvement, and high-efficiency clonal propagation (Wittmer et al., 2025).

### 4.6 *WIND* regulates cell dedifferentiation in wound response

Plant wounding serves as a potent trigger of tissue repair and organ regeneration, and the AP2/ERF transcription factor *WIND1* has been identified as a master regulator of wound-induced cell reprogramming and dedifferentiation. This factor is indispensable for conferring regenerative competence to explants during plant tissue culture (Iwase et al., 2015). Upon mechanical injury, plants rapidly initiate a cellular dedifferentiation program that reverts differentiated somatic cells to a pluripotent state, thereby establishing a prerequisite for subsequent regeneration. Despite the inherent robust regenerative capacity of plants in response to wounding, the primary signaling molecules and underlying regulatory mechanisms that initiate this dedifferentiation and regeneration cascade have long remain largely unknown. In 2024, researchers identified *Regeneration factor 1* (*REF1*) as the primary injury-responsive signaling molecule that initiates plant wound regeneration. Furthermore, they systematically delineated the signal transduction network through which *REF1* orchestrates tissue repair and organ regeneration in wounded plants (Yang et al., 2024).

## 5 Harnessing the DRs to overcome genotype dependence in genetic transformation

Accumulating evidence indicates that the ectopic overexpression of specific plant DRs can drastically enhance plant regeneration capacity and even trigger *de novo* organogenesis that rarely occurs under natural conditions (Lowe et al., 2016; Debernardi et al., 2020; Wang et al., 2022; Yu et al., 2024). For instance, the constitutive overexpression of *MdBBM1* has substantially improved transformation and regeneration efficiency in apples, yielding phenotypically normal transgenic plants and thus providing a novel strategy by which to surmount long-standing barriers in apple genetic engineering (Chen J. et al., 2022). Traditional maize transformation systems are constrained by a narrow germplasm range amenable to genetic engineering, which has become a major bottleneck in maize functional genomics research and crop genetic improvement. Through forward genetic screening, researchers have identified *Wox2a* as a candidate gene capable of triggering somatic embryogenesis, embryogenic callus formation, and subsequent plant regeneration in maize (McFarland et al., 2023). Furthermore, accumulating evidence indicates that *Arabidopsis AtWOX14* and its rice ortholog *OsWOX13* can significantly augment the regenerative capacity of their respective host species.

Lowe et al. (2016) was the first to demonstrate that the combinatorial expression of *BBM* and *WUS2* genes dramatically boosts maize transformation efficiency, elevating rates to 50% even in previously low-efficiency genotypes (Lowe et al., 2016). Leveraging these two regulators, researchers have successfully regenerated transgenic plants from mature embryo and leaf explants of maize—tissues that were previously recalcitrant to genetic transformation. The synergistic pro-regeneration effect of *ZmBBM* and *ZmWUS2* has also been validated in other recalcitrant crops, including sorghum, indica rice, and sugarcane, leading to significant improvements in their transformation efficiency (Lowe et al., 2016). In a more recent study, the coexpression of *HvWUS* and *HvBBM2* in barley was also shown to drastically enhance callus regeneration and transformation efficiency, effectively breaking through the genotype restrictions that had hindered barley biotechnology for decades (He et al., 2025).

Beyond the *BBM/WUS* module, the chimeric gene *TaGRF4-TaGIF1* has exhibited broad-spectrum efficacy in boosting regeneration and transformation efficiency when used for co-transformation, with positive outcomes reported in wheat, rye, rice, and citrus (Debernardi et al., 2020). Ectopic overexpression of *AtGRF5* in sugar beet calli accelerates shoot organogenesis and markedly improves transformation efficiency, enabling the generation of stable transformants in genotypes that were previously deemed recalcitrant (Kong et al., 2020). Similarly, the overexpression of *AtGRF6* and *AtGRF9* exerts a positive regulatory effect on transgenic callus proliferation in rapeseed. The *GRF5* gene has also been shown to enhance transformed cell proliferation and promote transgenic plant regeneration in diverse dicot crops, including soybean, sunflower, and watermelon (Kong et al., 2020). In maize, heterologous expression of the *AtGRF5* ortholog significantly elevates transformation efficiency, with regenerated transgenic plants exhibiting normal fertility (Kong et al., 2020). Most recently, Swinnen et al. (2025) reported that the overexpression of the SiGRF-SiGIF fusion protein in tomato significantly shortens the shoot regeneration cycle in callus cultures and doubles the number of regenerated shoots relative to control groups (Swinnen et al., 2025). Moreover, research has shown that *Agrobacterium*-mediated co-transformation of the fusion protein ZmBBM-ZmWUS2 and TaGRF4-TaGIF1 into mature embryo-derived calli of the wheat cultivar Kenong 199 increased the transformation efficiency of wheat mature embryos by up to 19.4%. This breakthrough has overcome a long-standing bottleneck that restricted wheat genetic transformation exclusively to immature embryos (Zhou et al., 2024).

## 6 Pleiotropic defects of DRs in plant genetic transformation

While DRs have emerged as powerful tools to overcome genotype-dependent recalcitrance in plant genetic transformation, their ectopic expression often causes severe pleiotropic developmental defects by disrupting finely regulated cell fate determination programs inherent to normal plant development. *WUS* and its grass-specific paralog *WUS2* maintain the SAM stem cell niche through the *WUS-CLAVATA3* negative feedback loop and the modulation of cytokinin signaling, but their misexpression disrupts meristem homeostasis, leading to abnormal vegetative and reproductive development including fasciated shoots, excess tillers, sterile floral organs, and impaired male gametogenesis (Leibfried et al., 2005). The AP2/ERF transcription factor *BBM* drives somatic embryogenesis by activating the *LAFL* regulatory network (Horstman et al., 2017), and the co-expression of *BBM* and *WUS2* synergistically boosts regeneration efficiency but also triggers severe developmental abnormalities such as dwarfism and complete sterility in transgenic plants (Lowe et al., 2016). The *GRF-GIF* module regulates cell proliferation and organ growth via chromatin remodeling and cell cycle control, with its ectopic activity leading to uncoupled cell proliferation without concomitant cell expansion, resulting in hyperplastic, reduced-size organs with altered cellular architecture (Kim et al., 2003; Horiguchi et al., 2005). Meanwhile, *WIND1* promotes wound-induced cell plasticity and dedifferentiation, and its constitutive overexpression induces ectopic callus formation on unwounded plant tissues, which severely impairs normal plant growth and development (Iwase et al., 2011). Collectively, while these DRs strongly enhance plant regeneration and transformation efficiency, their deleterious pleiotropic effects represent a major obstacle to their practical application in crop genetic engineering. A key challenge in plant biotechnology is thus to exploit the potent pro-regenerative potential of DRs while minimizing their adverse phenotypic effects—a goal critical for advancing crop genetic transformation technology.

## 7 Precision control of DRs in recalcitrant crop species

In 2025, Guo et al. established a streamlined and highly efficient co-transformation system for recalcitrant wheat cultivars (Guo et al., 2025). By delivering the TaGRF4-TaGIF1 fusion protein via co-transformation, they successfully achieved genetic transformation in two notoriously recalcitrant wheat cultivars, Aikang 58 and Xinong 979. Notably, the *TaGRF4-TaGIF1* transgene can be precisely excised from the genome of transgenic or gene-edited wheat plants via a recombinase-mediated system, thereby effectively circumventing the pleiotropic adverse effects associated with the constitutive expression of this DR chimera (Guo et al., 2025). In the same year, Hayta et al. (2025) developed and optimized a robust *Agrobacterium*-mediated transformation system for wheat, leveraging the *TaGRF4-TaGIF1* fusion gene as a regeneration booster (Hayta et al., 2025). A key optimization was the replacement of the constitutive *ZmUbi* promoter with the *ZmPLTP* promoter, which exhibits strong tissue-specific activity in embryogenic calli while maintaining minimal expression levels in mature plant tissues. Experimental data confirmed that, under the control of the *ZmPLTP* promoter, *TaGRF4-TaGIF1* exerted a potent pro-regenerative effect during the critical callus differentiation phase, thus ensuring high transformation efficiency. Meanwhile, the transgene remained transcriptionally silent during the subsequent vegetative and reproductive growth of the plants, which markedly reduced the incidence of deleterious phenotypic alterations. Transgenic wheat plants derived from this system displayed tiller numbers, flowering times, and fertility comparable to those of non-transgenic wild-type controls, confirming their normal growth and developmental patterns. Furthermore, the researchers incorporated a heat-inducible Cre/loxP recombination system into the transformation vector. Following callus regeneration and shoot formation, a simple heat treatment triggers the expression of Cre recombinase, which in turn precisely excises the *TaGRF4-TaGIF1* gene cassette from the host genome. This “burn-after-reading” strategy enables the efficient production of transgenic or gene-edited plants that are completely free of residual regeneration regulators (Hayta et al., 2025).

Conventional plant genetic transformation systems rely predominantly on *in vitro* tissue culture; however, transformation and regeneration efficiencies vary drastically across plant species, genotypes, and explant types, with many taxa remaining recalcitrant to such approaches. In contrast, plants inherently exhibit robust regenerative competence in response to mechanical wounding, a process that induces the expression of the key transcription regulator *WIND1*. A well-characterized downstream target of *WIND1* is *Enhancer of shoot regeneration 1* (*ESR1*), the promoter of which is specifically bound and activated by *WIND1* during the early wound response (Iwase et al., 2015). In 2025, Kshetry et al. capitalized on this wound-responsive regulatory cascade by placing the plant DR gene *isopentenyl transferase* (*ipt*) under the transcriptional control of the *ESR1* promoter (Kshetry et al., 2025) (**Figure 1I**). This design ensured that *ipt* expression was strictly confined to *WIND1*-activated cells at wound sites, thus limiting its pro-regeneration activity to the site of injury and regeneration. Using this system, they successfully generated *PDS* gene knockout mutant shoots directly from non-meristematic internodal tissues of *Nicotiana benthamiana*, bypassing *in vitro* tissue culture entirely. Notably, the system also exhibited efficacy in *Solanum* species and soybeans, demonstrating its broad applicability across diverse plant taxa. The core innovation of this strategy lies in the spatiotemporal restriction of DR expression to wound sites and the regeneration phase, which minimizes the pleiotropic side effects associated with the constitutive overexpression of such factors. Furthermore, integration of this wound-driven regeneration system with the CRISPR/Cas9 gene-editing system enables concurrent gene editing and organ regeneration, substantially reducing or even eliminating the requirement for labor-intensive *in vitro* tissue culture procedures (Kshetry et al., 2025). Collectively, this approach represents a groundbreaking strategy for overcoming long-standing bottlenecks in plant biotechnology, with the potential to accelerate the generation of transgenic and gene-edited plants while reducing reliance on traditional tissue culture-based workflows.

Recent research has elucidated the molecular mechanism of REF1-mediated wound regeneration: upon cellular damage in plants, the receptor kinase PORK1 (PEPR1/2 ORTHOLOG RECEPTOR-LIKE KINASE 1) recognizes *REF1* as the primary wound-signaling molecule. This receptor-ligand interaction triggers the expression of the master regulatory factor *WIND1*, thereby initiating downstream signaling cascades governing tissue repair and *de novo* organ regeneration. Furthermore, *WIND1* binds to the promoter region of the *REF1* precursor gene, upregulating its transcription and promoting the production of additional REF1 peptides. This positive feedback loop amplifies the *REF1*-mediated wound signal, which in turn modulates regenerative processes via plant cytokinin signaling pathways (Yang et al., 2024). Notably, the regeneration factor *REF1* is evolutionarily conserved across the plant kingdom, with orthologous peptides and their cognate receptors identified in nearly all dicotyledonous and monocotyledonous species examined to date. Exogenous application of REF1 has been shown to markedly enhance regeneration capacity and genetic transformation efficiency in tomato, soybean, wheat, and maize plants. This seminal study represents a major breakthrough in the elucidation of the molecular mechanisms underlying plant wound responses. The identification of *REF1* as the key wound-signaling molecule that orchestrates regenerative programs resolves a long-standing scientific conundrum in plant developmental biology. Beyond its theoretical significance, this discovery provides a practical and broadly applicable strategy for addressing critical bottlenecks in crop biotechnology, including low transformation efficiency and strong genotype dependence—two major challenges that have long hindered progress in plant breeding and genetic engineering.

In another line of investigation, exogenous application of adenosine monophosphate (AMP) was shown to markedly improve shoot regeneration efficiency from non-pluripotent callus during *Arabidopsis thaliana* leaf tissue culture. AMP treatment also enhanced protoplast proliferation, callus greening, and adventitious root formation in cabbage and tomato plants. Gene expression profiling revealed that AMP exerts its pro-regeneration effects by activating the *PLT* signaling pathway during plant organogenesis, indicating that AMP acts as a positive small-molecule regulator of *PLT*-mediated regeneration (Lee et al., 2023). These results suggest that supplementation with an optimal concentration of AMP during the callus proliferation phase can enhance tissue regeneration capacity across a broad range of plant species, providing a simple and scalable strategy to boost transformation efficiency in recalcitrant crops.

## 8 Conclusions and perspectives

Efficient and stable genetic transformation and gene editing represent pivotal bottlenecks in plant molecular design breeding, with their efficacy being tightly contingent on the regenerative capacity of explants tissues—a trait severely constrained by plant genotype (Lowe et al., 2016; Debernardi et al., 2020; Wang et al., 2022). Although a suite of DR-encoding regulatory genes (*BBM*, *WUS*, *WOX5*, *GRF-GIF* module, and *LAX1*) have been characterized and shown to boost transformation efficiency in several model crop species, most elite cultivated varieties—particularly commercially deployed cultivars—exhibit poor somatic embryogenesis or organogenesis capabilities. The performance of these regulatory genes is highly context-dependent across diverse genetic backgrounds, precluding the development of a universal and robust solution for all crop species. Thus, it is imperative to conduct systematic investigations into regeneration-related regulatory genes with broad-spectrum applicability across plant taxa. Specifically, dissecting their dose-response dynamics, spatiotemporal expression patterns, and interactive mechanisms with endogenous hormonal signaling pathways will lay a theoretical foundation for engineering precise “regeneration switches” that can be activated transiently during transformation and inactivated thereafter.

Conventional transformation protocols rely on the dedifferentiation and subsequent redifferentiation of explants tissues—processes that are inherently time-consuming, labor-intensive, and prone to somaclonal variation (Cao et al., 2023a). In recent years, non-tissue culture-based transformation strategies have emerged as promising alternatives, including *in planta* injection, CDB delivery, and virus-mediated vector systems. While these approaches circumvent the need for tedious *in vitro* procedures, they currently face significant technical constraints, including suboptimal DNA delivery efficiency, variable host species susceptibility to *Agrobacterium* infection, and challenges in achieving stable, heritable transgene integration in the germline. Consequently, their adoption has been limited to proof-of-concept demonstrations in select model species rather than broad application across diverse plant taxa. To advance the field of plant genetic transformation, future research should prioritize three key areas: (1) the systematic characterization of target crop species to elucidate the molecular basis of transformation recalcitrance, encompassing genetic background, innate regeneration capacity, and *Agrobacterium* compatibility. (2) the identification and functional validation of novel broad-spectrum DRs and small-molecule regulators (e.g., REF1 and AMP) that enhance regeneration without inducing severe pleiotropic effects; and (3) the synergistic integration of DR-mediated in planta regeneration with streamlined, culture-free DNA delivery modalities. A particularly promising avenue involves the precise spatiotemporal control of morphogenic DR genes through tissue-specific, inducible, or wound-responsive promoters, or via auto-excision cassettes—coupled with innovative delivery systems such as direct meristem injection or engineered viral vectors. This integrated approach may enable the development of robust, efficient, and genotype-flexible transformation platforms that facilitate on-demand genome editing in recalcitrant elite commercial cultivars.

Notably, current genotype-flexible transformation systems have been validated predominantly in cereals (wheat, maize, rice) and select legumes, with limited exploration in oilseed crops, horticultural species, and woody perennials. Moreover, elite commercial varieties consistently exhibit lower transformation competence than model genotypes, underscoring the persistent challenge of genotype dependency even within optimized protocols. Overcoming these species- and genotype-specific barriers will require interdisciplinary research that combines plant developmental biology, epigenetics, synthetic biology, and vector engineering. Ultimately, the development of truly genotype-flexible plant genetic transformation technologies will unlock the full potential of CRISPR-mediated genome editing and synthetic biology for crop improvement, enabling the rapid development of climate-resilient, high-yield, and nutrient-dense crop varieties to address global food security challenges.
